# Modified Formulations of Silicate-Based Cements: Comparative Study of Physicochemical Properties

**DOI:** 10.3390/ma19061083

**Published:** 2026-03-11

**Authors:** Mirlyn de Souza Dias, Raimundo Sales de Oliveira Neto, Marcelo Antônio Santos da Silva, Suyane Maria Luna-Cruz, Murilo Priori Alcalde, Rodrigo Ricci Vivan, Antônio Sérgio Bezerra Sombra, Marco Antônio Húngaro Duarte, Pierre Basilio Almeida Fechine, Bruno Carvalho de Vasconcelos

**Affiliations:** 1 Postgraduate Program in Dentistry, Federal University of Ceará, 1273 Monsenhor Furtado St., Rodolfo Teófilo, Fortaleza 60455-730, CE, Brazil; mirlyn.dias@gmail.com (M.d.S.D.); suyane.cruz@professor.unifametro.edu.br (S.M.L.-C.); 2 Department of Operative Dentistry, Endodontics, and Dental Materials, Bauru School of Dentistry, University of São Paulo, Bauru 17012-191, SP, Brazil; raimundoneto@usp.br (R.S.d.O.N.); malcalde@fob.usp.br (M.P.A.); rodrigo.vivan@fob.usp.br (R.R.V.); mhungaro@fob.usp.br (M.A.H.D.); 3 Telecommunications and Materials Science & Engineering Laboratory, Federal University of Ceará, Fortaleza 60440-900, CE, Brazil; marceloassilva@gmail.com (M.A.S.d.S.); asbsombra@gmail.com (A.S.B.S.); 4 Dentistry Course, UniFametro, Fortaleza 60010-280, CE, Brazil; 5 Group of Chemistry of Advanced Materials (GQMat), Department of Analytical Chemistry and Physical-Chemistry, Federal University of Ceará, Fortaleza 60440-900, CE, Brazil; fechine@ufc.br

**Keywords:** Endodontics, silicate cement, physicochemical properties, biocompatible materials

## Abstract

**Highlights:**

**What are the main findings?**

- The experimental cements exhibited adequate physicochemical performance.
- The radiopacity exceeded the ISO requirements without the need for radiopacifiers.
- The setting time improved compared with previous formulations.
- The solubility remained within acceptable limits.
- An alkaline pH and sustained calcium/strontium ion release were observed.

**What are the implications of the main findings?**

- Calcium silicate-based cements with zirconium or strontium phosphate may reduce the need for radiopacifiers.
- Formulations show potential as alternatives to commercial materials.
- The results support future biological and clinical investigations.

**Abstract:**

This study aimed to prepare experimental calcium silicate repair cements (ERCs) incorporating zirconium (Ca_3_ZrSi_2_O_9_; CZS) or strontium substitution (Sr_5_(PO_4_)_2_SiO_4_; SPS), and to compare their physicochemical properties with white MTA-Angelus (WMTA), grey MTA-Angelus (GMTA), and Biodentine (BD). After synthesizing the CZS and SPS phases, powder–liquid cements were formulated. The setting time and radiopacity were assessed according to ISO 6876/12 and ASTM C266, the volumetric solubility by micro-CT, the pH by a pH meter, and the calcium/strontium ion release by FAAS/ICP-OES. Data were analyzed using ANOVA and Tukey’s tests (5%). The initial setting time was 11 min for SPS and 6 min for CZS (*p* < 0.05), while the final setting was significantly longer for SPS (49 min). Both ERCs showed radiopacity above the 3.0 mm Al minimum, with higher values for CZS (4.58 mm Al). The solubility remained controlled, with CZS presenting the highest value (3.09%). Both materials exhibited an alkaline pH, peaking at 24 h (CZS: 9.70; SPS: 10.04) and decreasing until 168 h (CZS: 7.80; SPS: 8.31). Sustained ionic release was observed: CZS showed intermediate calcium release (25.96 mg/L at 3 h), whereas SPS displayed lower values (10.95 mg/L at 168 h), without significant difference from WMTA (*p* > 0.05). Under these conditions, the experimental ERCs demonstrated adequate physicochemical performance comparable with commercial materials.

## 1. Introduction

Endodontics is one of the dental specialties that most benefits from the development of new biomaterials. Procedures such as pulp capping, sealing of perforations and root resorptions, revascularization, apexification, and retrofilling in periradicular surgery depend on the selection of repair materials with adequate physicochemical and biological properties [1]. Clinical success is related not only to the sealing ability but also to biocompatibility, antimicrobial activity, and stimulation of mineralized tissue formation [2].

Mineral Trioxide Aggregate (MTA), introduced in 1993, became a reference material due to its bioactivity and ability to induce cell differentiation and mineral deposition [3]. However, limitations such as difficult manipulation, prolonged setting time, low radiopacity, risk of tooth discoloration, and the presence of toxic components encouraged the search for new materials [1,4]. To overcome these limitations, new calcium silicate-based cements were developed, including Biodentine (BD; Septodont, Saint-Maur-des-Fossés, Cedex, France), which is composed of tricalcium silicate, zirconium oxide, and calcium carbonate. This material offers improved handling [5] and reduced setting time [6] without compromising biocompatibility [7]. Nevertheless, no cement exhibits all ideal properties, justifying the search for alternative formulations.

Calcium silicate cements stand out for their favorable interaction with periradicular tissues, mainly due to the release of calcium and silicon ions capable of stimulating cell differentiation and mineral deposition [8]. Additionally, incorporating new elements into bioceramic cement formulations has proven a promising strategy. Zirconium enhances the mechanical strength and chemical stability and shows satisfactory biocompatibility [9]. Strontium, a physiologically calcium-like element, exerts multiple beneficial effects on bone metabolism, including stimulation of bone formation, reinforcement of bone structure, and both anabolic and anticatabolic effects, resulting in increased bone mass [10,11,12]. Moreover, Sr^2+^ ion release promotes apatite precipitation and increases the material’s bioactivity, biocompatibility, and mechanical strength [11,13]. In this context, experimental cements based on calcium zirconium silicate (Ca_3_ZrSi_2_O_9_; CZS) and strontium phosphate silicate (Sr_5_(PO_4_)_2_SiO_4_; SPS) have been investigated, demonstrating promising biocompatibility and physicochemical performance, although with limitations related to the setting time and solubility [11]. These formulations were selected because they are reproducible and not overly complex to synthesize, contain known biocompatible components, and are capable of acting as a source of the desired ions.

Thus, since the previous study performed by the same research group obtained favorable results when testing experimental repair cements (ERCs) based on CZS and SPS [11], this study aimed to synthesize these silicates once again; incorporate them into experimental repair cement with modified formulations; and evaluate the physicochemical properties of setting time, radiopacity, volumetric solubility, pH, and calcium/strontium ion release. Additionally, these properties were compared with those of the commercial cements MTA Angelus Grey (GMTA; Angelus Indústria de Produtos Odontológicos S/A, Londrina, Paraná, Brazil), MTA Angelus White (WMTA; Angelus Indústria de Produtos Odontológicos S/A), and Biodentine. The null hypothesis was that no statistically significant differences would be found between the experimental cements or between them and the commercial materials.

## 2. Materials and Methods

The study involved the synthesis of two silicates, namely, calcium zirconium silicate (CZS) and strontium phosphate silicate (SPS), obtained via a solid-state route as described below; both syntheses followed the protocol described by Aguiar et al. [11].

### 2.1. Synthesis of Calcium Zirconium Silicate (CZS)

CZS was obtained by mixing stoichiometric amounts of calcium oxide (CaO), zirconium oxide (ZrO_2_), and silicon oxide (SiO_2_) (Êxodo Científica Química Fina Indústria e Comércio Ltda., Sumaré, São Paulo, Brazil) and mechanically homogenized using a mortar and pestle before being transferred to ceramic crucibles. The reaction occurred in an oxidative atmosphere at 1350 °C in a resistive furnace for 8 h with a heating rate of 5 °C/min. The resulting sample was then ground and characterized.

### 2.2. Synthesis of Strontium Phosphate Silicate (SPS)

SrCO_3_ (Êxodo Científica), SiO_2_ (Êxodo Científica), and ammonium dihydrogen phosphate (NH_4_H_2_PO_4_, Neon Comercial, Sumaré, São Paulo, Brazil) were used as precursors. The oxides were mechanically homogenized using a mortar and pestle and transferred to ceramic crucibles. The reaction was conducted in an oxidative atmosphere at 1350 °C for 8 h with a 5 °C/min heating rate. The sample was then ground and characterized.

### 2.3. X-Ray Diffraction (XRD) Characterization

XRD analyses were performed at room temperature using a Bruker diffractometer (D8 Advance; Bruker do Brasil Ltda, Atibaia, SP, Brazil) with a CuKα tube (λ = 1.5418 Å) and LynxEye linear detector: 40 kV, 40 mA, and 2θ range 10–80° with 0.02° step. The crystalline phases of CZS and SPS after calcination were compared with patterns in the Inorganic Crystal Structure Database (ICSD).

### 2.4. Physicochemical Tests

SPS and CZS were used as the main components of experimental powder–liquid repair cements. Other substances were added to confer different physicochemical characteristics. Powders were manually mixed on a glass plate for 1 min at a 3:1 ratio (g/g) with a liquid primarily composed of distilled water. Commercial cements GMTA, WMTA, and BD were prepared according to manufacturer instructions. Their compositions are shown in Table 1. The physicochemical properties of both experimental and commercial cements were evaluated as follows.

#### 2.4.1. Setting Time

The setting time was determined according to ISO 6876/2012 [14] using ASTM C266-20 [15] as a reference for Gilmore needles. Type IV gypsum rings (10 mm inner diameter, 2 mm height) were immersed in deionized water for 24 h. Prepared cements (n = 3) were inserted into the rings and stored at 37 °C and 95 ± 5% humidity. After 180 s, measurements were taken using Gilmore needles: 113.4 g with a 2.0 mm tip for initial setting and 453.6 g with a 1.0 mm tip for final setting.

#### 2.4.2. Radiopacity

The radiopacity was assessed according to ISO 6876/2012 [14]. Metallic rings (n = 3, 10 mm diameter, 1 mm height) were filled with cements and incubated at 37 °C and 95 ± 5% humidity for three times the setting period. Specimens were radiographed alongside an aluminum step wedge (1–10 mm Al) on occlusal film (60 kV, 10 mA, 0.3 s, 30 cm). Images were digitized and analyzed in ImageJ 8.0, converting the radiographic density to mm Al [16].

#### 2.4.3. Volumetric Solubility

Considering the lack of a specific standardization norm, sample size calculation was performed for this analysis. For this purpose, the G*Power v3.1 for Mac software (Heinrich Heine University Düsseldorf) was used, selecting one-way ANOVA, employing the values available on Cavenago et al. [17] (SD = 0.77 for MTA Angelus, 3:1 ratio), and adopting a minimum detectable difference of 1.5, which yielded six specimens per group (power = 80%, α = 0.05). The solubility was evaluated by micro-computed tomography (SkyScan 1174v2; SkyScan, Kontich, Belgium). Acrylic blocks with standardized cavities (n = 6, 2 mm diameter, 3 mm depth) were filled, scanned after final setting (50 kV, 800 μA, voxel 14.1 μm, 1.1° step), and analyzed in CTan software (v.1.14.4; Bruker microCT, Kontich, Belgium) for the total volume. Samples were immersed in 15 mL deionized water at 37 °C for 168 h, dried, and rescanned. Solubility was expressed as the percentage of volume lost.

#### 2.4.4. pH and Calcium/Strontium Release

As same as for volumetric solubility, for pH and Ca^2+^/Sr^2+^ analysis was calculated using one-way ANOVA, using the G*Power v3.1 for Mac software and Vivan et al.’s [18] method (SD = 1.03 at 3 h), and adopting a detectable difference of 1.5, which resulted in ten specimens per group (power = 80%, α = 0.05). Freshly mixed cements were placed in polyethylene tubes (n = 10, 1 mm diameter, 10 mm length, one end sealed) and immersed in 10 mL deionized water (initial pH 7.11) at 37 °C and 95 ± 5% humidity. The tubes remained in water for 3, 24, 72, and 168 h, and were transferred to fresh water at each interval; tubes in water only and empty tubes were the controls. The pH was measured after 5 s of mechanical agitation using a calibrated digital pH meter (Micronal #371, São Paulo, Brazil). The calcium release was quantified by flame atomic absorption spectrometry (SpectraAA 220 FS; VARIAN, NJ, USA) with La(NO_3_)_3_ 10 g/L to remove alkali metal interferences (422.7 nm, 0.2 mm slit, 10 mA, 2.0 L/min acetylene). Strontium release was evaluated by inductively coupled plasma optical emission spectrometry (ICP-OES) (Optima 2100 DV, Perkin Elmer, Waltham, MA, USA) at Sr 407.771 nm.

### 2.5. Statistical Analysis

Data were tabulated and tested for normality using the Kolmogorov–Smirnov test, which indicated a normal distribution. Considering this, the statistical software GraphPad Prism 9.0 (GraphPad Software Inc., San Diego, CA, USA) was used to analyze the parametric data employing the one-way ANOVA and Tukey post hoc tests, with significance set at 5%.

## 3. Results

### 3.1. X-Ray Diffraction (XRD) Characterization Values

**Figure 1:**
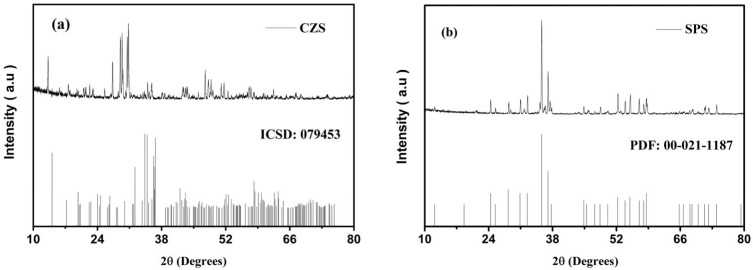
XRD pattern of the powder after sintering at 1350 °C for the CZS (**a**) and SPS (**b**) samples.

Figure 1 shows the XRD patterns of the CZS (1a) and SPS (1b) samples. In Figure 1a, the diffractogram obtained from the CZS synthesis (line graph) is compared with the ICSD standard pattern. All diffraction peaks of CZS were observed, confirming the formation of CZS through the calcination process used. In Figure 1b, the diffractogram for SPS synthesis is presented similarly. Two graphs are shown: the line graph represents the experimental measurement of SPS, and the other represents the standard SPS pattern from ICSD. All diffraction peaks corresponding to the SPS phases are present in the synthesized sample diffractogram.

### 3.2. Physicochemical Properties

Table 2 presents the results for the setting time, radiopacity, and solubility of the experimental (SPS and CZS) and commercial cements included in this study.

Regarding the initial setting time, the order observed was CZS < WMTA < GMTA < BD < SPS, with SPS (11.33 min), GMTA, and BD significantly higher than CZS (6 min) and WMTA (*p* < 0.05); CZS was the fastest-setting material. For the final setting time, the order was BD < WMTA < GMTA < CZS < SPS, with significant differences between SPS (49 min) and the other groups, and between BD (18 min) and the others (*p* < 0.05); BD had the fastest final setting.

**Figure 2:**
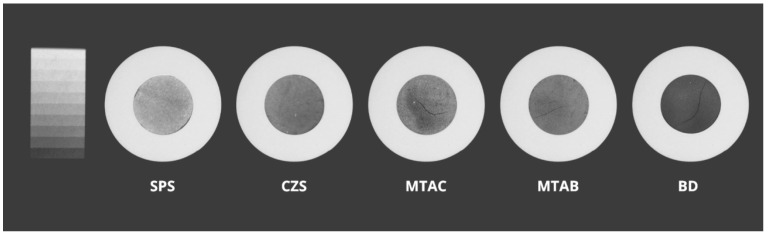
Visual comparison of the radiopacity of the tested cements in relation to the aluminum step wedge.

For the radiopacity, the order was SPS > CZS > GMTA = WMTA > BD, with SPS (4.58 mm Al) and BD (1.64 mm Al) differing significantly as the highest and lowest values, respectively (*p* < 0.05). Only SPS and CZS (3.18 mm Al) exceeded the ISO 6876/2012 minimum of 3.0 mm Al. Figure 2 allows visual comparison of the radiopacity differences between the materials.

**Figure 3:**
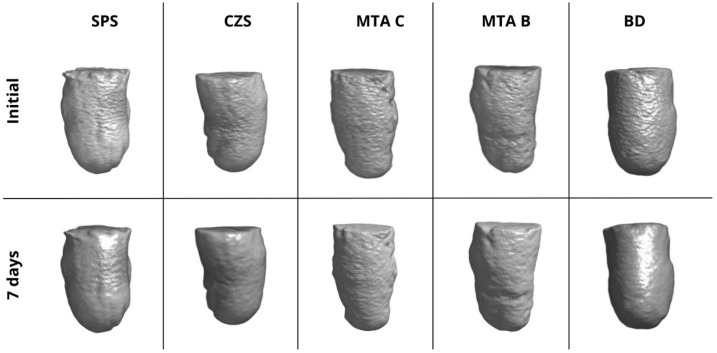
Three-dimensional reconstructions obtained by micro-computed tomography of the samples before immersion and after 7 days.

Regarding the volumetric solubility measured by micro-CT, the order was GMTA < WMTA < SPS < BD < CZS. SPS (2.28%) did not differ significantly from the other groups, while CZS (3.09%) had higher values, differing statistically from GMTA (1.21%) and WMTA (1.31%) (*p* < 0.05). Figure 3 shows 3D reconstructions of the samples before immersion and after 7 days.

The pH values are shown in Table 3. Both SPS and CZS showed a slight increase from 3 to 24 h followed by a decline, whereas commercial cements decreased progressively. At 3 h, the order was BD > GMTA > CZS > WMTA > SPS, with significant differences between SPS (9.01) and BD (10.52) and GMTA (10.11), and between CZS (9.59) and BD (*p* < 0.05). At 168 h, the order was WMTA > GMTA > SPS > BD > CZS, with CZS (7.80) differing significantly from the MTAs (*p* < 0.05).

For the ion release (Table 4), the overall order was BD > CZS > GMTA > WMTA > SPS, with BD showing the highest release at all time points (*p* < 0.05). Conversely, SPS showed the lowest release at all time points, significantly lower than GMTA, CZS, and BD at 3 h and 168 h (*p* <0.05). CZS had an intermediate pattern, differing only from GMTA at 3 h, 24 h, and 168 h. The within-group analysis showed no significant differences over time for CZS and WMTA; SPS differed at 24 h compared with the other time points (*p* < 0.05).

## 4. Discussion

Materials such as MTA-Angelus and Biodentine have become references due to their physicochemical properties, biocompatibility, bioactivity, and ability to induce mineralized tissue formation, but they still present limitations [19,20]. In this context, this study aimed to advance in the development of experimental cements using other types of silicates, with zirconium or strontium, along with the addition of auxiliary substances, aiming to overcome weaknesses of commercial cements and provide effective alternatives for clinical practice. The findings show that the modified ERCs prepared with the developed silicates exhibited properties close to those of commercial cements, with superior radiopacity and lower ion release; since some of these differences were significant, the null hypothesis was rejected.

Using the expected peaks after synthesis (ICSD) as the reference, it can be stated that the preparation of the silicates was successful; considering that the syntheses performed here respected all the parameters of the previous study [11], other analyses related to characterization were not necessary. Regarding this point, the previous study of the same research group, which tested materials similar to the ERCs evaluated here, suggested the need for modifications in their composition [11]. In this sense, calcium oxide and calcium carbonate were added to the powder. Calcium oxide was added as a setting accelerator and expansion inducer, promoting the formation of calcium hydroxide, which would facilitate the transition from the initial pasty consistency to a solid and stable structure over time [21,22]. Calcium carbonate, in turn, improved handling, flow, and facilitated material insertion, in addition to contributing to cement hydration [23,24]. In the liquid, water-soluble polymers and a setting accelerator were added to enhance the particle dispersion, suspension stability, reaction kinetics control, and flow, and to promote the initiation of the hydration reaction [25].

Regarding the findings, the setting times of the formulations tested showed a reduction when compared with those reported in the literature (SPS 11/49 min vs. 21/50 min; CZS 6/36 min vs. 31/68 min) [11], suggesting that the inclusions were favorable. Regarding the commercial cements, they showed values consistent with the literature [26,27,28], with both types of MTA characterized by a final setting time of approximately 30 min, while BD had a final setting time of about 18 min. Clinically, very short setting times can hinder material insertion; conversely, very long times may compromise material stability after insertion, promote dissolution of components by blood or tissue fluids, or cause tissue irritation with some degree of toxicity [29,30,31].

Concerning the radiopacity, the ERCs showed values above the ISO 6876/2012 minimum of 3.0 mm Al, even without adding any additional radiopacifier. This finding corroborates a previous study [11] and is due to the intrinsic presence of zirconium or strontium in the silicates, which increases radiographic density due to their high atomic mass (91.22 and 87.62, respectively). This result eliminates the need for adding radiopacifiers, such as bismuth oxide, which can cause tooth discoloration and interfere with the physicochemical properties of materials [32]. In the case of commercial cements, values below the minimum required by the standard were observed, namely, 2.94 mm Al for GMTA and WMTA and 1.64 mm Al for Biodentine, which are consistent with literature reports [28,33,34].

Regarding the volumetric solubility, micro-CT analysis was chosen, which is a methodology that provides greater precision in quantifying structural material loss compared with traditional gravimetric testing by avoiding misinterpretations associated with fluid absorption, which is a common phenomenon in hydraulic cements [17,35,36]. Clinically, low solubility ensures maintenance of sealing and cement stability, but it tends to be inversely proportional to ion release [29,37]. The ERCs tested here maintained solubility within the 3% limit, differing from previous findings where higher values were reported [11]. This can be attributed both to the presence of setting accelerators in the liquid and powder, which resulted in a shorter initial setting time, and to the presence of calcium oxide, which may have provided some expansion [21,22,38]. Another factor may be associated with the water-soluble polymers present in the liquid, which, due to their plasticizing and humidifying action, may have resulted in greater homogeneity, reducing porosity and increasing matrix cohesion [25,39].

It is natural for silicate reactions to form a fraction of calcium hydroxide, consequently releasing hydroxyl ions, raising the pH, and creating an alkaline environment with antimicrobial effects and favoring mineralized tissue deposition [40]. The results showed that BD exhibited a higher pH than the others during the first 3 h, consistent with the literature, which reports similar behavior for this material due to its high hydration rate and initial solubilization [11,29,41]. In the experimental cements, the pH increase observed at 24 h was largely due to the period of material solubilization; subsequently, there was a slight decline, remaining mildly alkaline, as described in previous studies [29,42]. It should be noted that the pH of tissue formation is only slightly alkaline, and cell growth is impaired at pH levels above 7.8 [43].

Additionally, the sustained release of calcium and strontium ions contributes specifically to the bioactivity of the material [10,43]. Calcium stimulates differentiation of osteoblasts, cementoblasts, and pulp cells, and induces the expression of signaling proteins such as osteocalcin and alkaline phosphatase, promoting deposition of mineralized matrix [8,44]. Strontium, in turn, has a dual effect, stimulating adhesion, proliferation, and differentiation of osteoblasts, while inhibiting osteoclastic activity, favoring mineralized tissue formation and reducing bone resorption [10].

Regarding calcium release, BD was also the material showing the highest ionic release compared with the experimental cements and the other commercial cements tested, which corroborates previous studies highlighting its capacity for more intense and sustained calcium release [29,45]. Among the experimental cements, SPS showed a calcium release pattern similar to MTA Angelus White (WMTA), whereas CZS showed behavior closer to MTA Angelus Gray (GMTA), indicating that the ERC formulations satisfactorily replicated the characteristics of the reference commercial cements.

When compared with results obtained by Aguiar [11], the proposed modifications reduced the solubility, accelerated the setting, and maintained an adequate pH and ionic release. These attributes highlight the potential of modified cements as innovative alternatives for endodontic use. Undoubtedly, there are still aspects to improve in the material composition; however, it is important to note that statistical differences may not always reflect clinical relevance.

From a future perspective, the developed experimental cements present promising and improved characteristics when compared with those introduced previously [11]. Nevertheless, despite encouraging in vitro results, further studies, including biological analyses and clinical trials, are recommended to validate the safety and efficacy of these newly tested formulations.

## 5. Conclusions

Under the conditions of this study, it can be concluded that the developed experimental reparative cements demonstrated physicochemical performance close to that of the commercial cements tested, although additional adjustments and biological investigations are necessary to consolidate their use in clinical practice.

## Figures and Tables

**Table 1:** Chemical composition of the cements used.

Material	Powder	Liquid
Experimental Strontium Phosphate Silicate Cement (SPS)	Strontium phosphate silicate	Ultrapure water
	Calcium oxide	Water-soluble polymers
	Calcium carbonate	Setting accelerator
Experimental Calcium Zirconium Silicate Cement (CZS)	Calcium zirconium silicate	Ultrapure water
	Calcium oxide	Water-soluble polymers
	Calcium carbonate	Setting accelerator
MTA-Angelus Cinza (GMTA; Angelus Ind. Com. LTDA) #79141	Tricalcium silicate	Distilled water
	Dicalcium silicate	
	Tricalcium aluminate	
	Calcium oxide	
	Tetracalcium aluminoferrite	
	Bismuth oxide	
MTA-Angelus Branco (WMTA; Angelus Ind. Com. LTDA) #66086	Tricalcium silicate	Distilled water
	Dicalcium silicate	
	Tricalcium aluminate	
	Calcium oxide	
	Calcium tungstate	
Biodentine (BD; Septdont) #B33283	Tricalcium silicate	Dihydrated calcium chloride
	Zirconium oxide	Aerosil
	Calcium oxide	Purified water
	Calcium carbonate	
	Yellow pigment	
	Red pigment	
	Brown iron oxide	

**Table 2:** Values (mean and standard deviation) of the cements for setting time (minutes), radiopacity (mm Al), and solubility (%).

Groups	Setting Time				Radiopacity		Solubility	
	Initial		Final					
	Mean	SD	Mean	SD	Mean	SD	Mean	SD
SPS	11.33 ^b^	0.58	49.00 ^c^	1.00	4.58 ^a^	0.17	2.28 ^abc^	0.85
CZS	6.00 ^a^	1.00	36.33 ^b^	2.31	3.18 ^b^	0.15	3.09 ^c^	0.95
GMTA	9.33 ^b^	0.58	30.33 ^b^	4.04	2.94 ^b^	0.20	1.21 ^a^	0.61
WMTA	6.33 ^a^	0.58	29.67 ^b^	2.08	2.94 ^b^	0.34	1.31 ^ab^	0.35
BD	10.33 ^b^	1.53	18.00 ^a^	2.65	1.64 ^c^	0.35	2.80 ^bc^	1.07

Different lowercase superscript letters indicate significant differences between the experimental groups.

**Table 3:** Values (mean and standard deviation) of the pH of the evaluated cements at different time points.

Groups	3 h		24 h		72 h		168 h	
	Mean	SD	Mean	SD	Mean	SD	Mean	SD
SPS	9.01 ^c,B^	0.558	10.04 ^a,A^	0.291	9.71 ^a,A^	0.430	8.31 ^abc,C^	0.427
CZS	9.59 ^bc,A^	0.527	9.70 ^ab,A^	0.181	8.44 ^c,B^	0.375	7.80 ^c,C^	0.249
GMTA	10.11 ^ab,A^	0.714	9.99 ^a,A^	0.541	9.22 ^ab,B^	0.581	8.42 ^ab,C^	0.636
WMTA	9.54 ^bc,A^	0.508	9.17 ^b,AB^	0.626	8.83 ^bc,B^	0.685	8.69 ^a,B^	0.469
BD	10.52 ^a,A^	0.579	9.68 ^ab,B^	0.639	8.38 ^c,C^	0.424	8.07 ^bc,C^	0.351

^a,b,c^ Different lowercase superscript letters indicate significant differences between the experimental groups according to ANOVA and Tukey’s tests (*p* < 0.05). ^A,B,C^ Different uppercase superscript letters indicate significant differences within each group across the different time points according to ANOVA and Tukey’s tests (*p* < 0.05).

**Table 4:** Values (mean and standard deviation) of the ion release (mg/L) of the evaluated cements at different time points.

Groups	3 h		24 h		72 h		168 h	
	Mean	SD	Mean	SD	Mean	SD	Mean	SD
SPS	7.28 ^d,B^	2.430	10.95 ^c,A^	1.717	8.37 ^c,B^	1.366	7.19 ^c,B^	0.367
CZS	25.96 ^b,A^	11.860	21.37 ^b,A^	4.297	21.63 ^b,A^	4.625	17.43 ^b,A^	4.835
MTA C	18.77 ^bc,A^	6.390	16.59 ^bc,AB^	4.961	12.52 ^c,B^	3.457	13.17 ^b,AB^	5.020
MTA B	9.64 ^cd,A^	3.363	12.56 ^c,A^	6.239	9.95 ^c,A^	6.076	7.56 ^c,A^	1.314
BD	49.53 ^a,A^	9.544	30.30 ^a,C^	6.201	32.53 ^a,BC^	4.953	40.11 ^a,B^	6.635

^a,b,c,d^ Different lowercase superscript letters indicate significant differences between the experimental groups according to ANOVA and Tukey’s tests (p < 0.05). ^A,B,C^ Different uppercase superscript letters indicate significant differences within each group across the different time points according to ANOVA and Tukey’s tests (*p* < 0.05).

## Data Availability

The original contributions presented in this study are included in the article. Further inquiries can be directed to the corresponding author.

## Abbreviations

The following abbreviations are used in this manuscript:

MTA	Mineral Trioxide Agreggate
BD	Biodentine
CZS	Calcium zirconium silicate
SPS	Strontium phosphate silicate
WMTA	White MTA-Angelus
GMTA	Gray MTA-Angelus
XRD	X-Ray diffraction
ICSD	Inorganic Crystal Structure Database
ISO	International Organization for Standardization
ASTM	American Society for Testing Materials
SD	Standard deviation
ICP-OES	Inductively coupled plasma optical emission spectrometry
